# Brain metastases from hepatocellular carcinoma: recent advances and future avenues

**DOI:** 10.18632/oncotarget.15730

**Published:** 2017-02-25

**Authors:** Shanshan Wang, Anqiang Wang, Jianzhen Lin, Yuan Xie, Liangcai Wu, Hanchun Huang, Jin Bian, Xiaobo Yang, Xueshuai Wan, Haitao Zhao, Jiefu Huang

**Affiliations:** ^1^ Department of Liver Surgery, Peking Union Medical College Hospital, Chinese Academy of Medical Sciences and Peking Union Medical College, Beijing, China; ^2^ Center of Translational Medicine, Peking Union Medical College Hospital, Chinese Academy of Medical Sciences and Peking Union Medical College, Beijing, China

**Keywords:** brain metastases, hepatocellular carcinoma, radiotherapy, targeted therapy, immunotherapy

## Abstract

The incidence of brain metastases from hepatocellular carcinoma (BMHCC) is becoming more frequent than that of the past as a result of prolonged survival of patients with HCC. Compared with brain metastases from other types of cancer, BMHCC tends to exhibit a high incidence of intracerebral hemorrhage (ICH) and poor liver function. Unfortunately, the prognosis is extremely poor for patients with BMHCC owing to the limited treatment selection. Currently, optimal treatment requires multidisciplinary approaches including surgery, whole-brain radiation therapy and stereotactic radiosurgery. Besides these traditional approaches, novel treatments such as target therapy and immunotherapy provide an opportunity to improve the survival of these patients. This review provides an overview of the incidence, characteristics, prognosis, and current and potential future management strategies for BMHCC.

## INTRODUCTION

Brain metastases from hepatocellular carcinoma (BMHCC) are extremely rare, occurring in approximately 1% of HCC patients [1]. Previously, clinicians paid little attention to this clinical scenario because symptoms suggestive of metastasis were rarely observed due to the poor overall prognosis of HCC patients [2]. However, as a result of recent progress in both the diagnosis and treatment of HCC, prognosis HCC have improved much, brain metastases from HCC (BMHCC) are now being diagnosed more frequently [3, 4]. Disappointingly, the outcome of BMHCC has not substantially changed despite advances in therapeutic options for BM and HCC. The substantial burden of morbidity and mortality associated with these metastases has motivated research and technological innovation over the past two decades. The purpose of this review is to highlight emerging data on BMHCC epidemiology and modalities used in the management of BMHCC, briefly outline current treatment approaches with an emphasis on novel and emerging therapies, and discuss areas of future research focus.

## INCIDENCE OF BRAIN METASTASES IN HCC PATIENTS

Brain metastases (BM) are the most common and devastating neurologic complications of systemic cancer and occur in 20% to 40% of advanced-stage cancers [5, 6]. BM mainly occurs in patients with lung cancer (40-50%), breast cancer (15-25%), and melanoma (5-20%) [7–9] and BM arising from HCC is extremely rare, with a reported incidence ranging from 0.2% to 2.2% [10–15]. As stated by Jiang et al. [3], these figures probably underestimate the scale of the problem, and several autopsy series suggest that the underlying incidence is probably around 2.0-7.7% [13]. Nevertheless, the incidence of BMHCC is much lower than that of HCC metastasis to other organs, which may be due to the low affinity of HCC for the central nervous system (CNS) and the rapid disease course and short survival time of patients with HCC, which decreases the likelihood brain metastases [16, 17]. However, the incidence appears to have increased over past decades (Table 1). The first large retrospective study published in 1998 by Kim et al. found that only seven of 3,100 HCC patients had intracranial metastases [11]. On the basis of data from 10,615 patients recorded in the Yonsei University Health System between 1973 and 2001, Lim et al. suggested that the incidence had increased to 1.1% [12]. Similarly, Shao et al. assessed 158 patients with advanced HCC who were treated with antiangiogenic targeted therapy at National Taiwan University Hospital between 2005 and 2009, and reported an increase in incidence to 7% [18]. This progressive increase is probably due to longer survival of patients with HCC [7] and increased utilization of sensitive detection methods, particularly MRI, which is currently used to assess approximately 64% of patients with cancer compared with only 2% of similar patients groups 20 years ago [19, 20]. Given these data, BM can no longer be regarded as a rare event in HCC patients.

**Table 1:** Summary of selected reported case-series of BMHCC

Study (year)	Country	Years included	HCC cases	Cases (n)	Inc (%)	Male (%)	Median age (years)	Single BM (%)	ICH (%)	Diagnosis tool	Time since HCC diagnosis (months)	ECM (%)	Treatment modality(%)	OS (months)	Significant prognostic factors
Kim et al.11(1998)	Korea	1987-1991	3100	7	0.23	85.7	56	NA	57.1	CT or/and MRI	15.3	Lung:28.6	RT	3.9	NA
Chang et al.49(2004)	Taiwan	1986-2002	NA	45	NA	88.9	NA	58	40	CT or/and MRI	10.5	NA	SR and/or RT	SR/RT:>4 SC:<1	Single lesion
Natsuizak et al.13(2005)	Japan	1995-2001	482	5	1.04	NA	62	NA	NA	NA	NA	NA	RT,SR	NA	NA
Seinfeld et al.14 (2006)	USA	1992-2004	NA	3	NA	57.8	33.3	NA	NA	CT or/and MRI	NA	NA	SR+SRT;SR	2	NA
Chen et al.16 (2007)	Taiwan	1993-2003	15,008	32	0.21	NA	32	90	NA	CT or/and MRI	NA	NA	NA	3.3	NA
Hsieh et al. 52 (2009)	Taiwan	NA	NA	42	NA	81.0	56	67	43	CT or/and MRI	15.4	ALL:81.0 Lung:47.6 Bone:14.2 Lymph nodes:19.0 others:14.2	SC/symptomatic therapy:33.3 WBRT:52.4 SR+WBRT: 9.6 SR: 4.8	ALL:1.2 ICH:1.0 no-ICH:1.3	ICH did not influence
Chan et al. 10 (2009)	Taiwan	1988-2008	2245	28	1.2	NA	NA	89	NA	CT or/and MRI	NA	NA	SR:29.1	6.1	NA
Choi et al.33 (2009)	Korea	1995-2006	6919	62	0.9	75.8	54	62.9	55	CT or/and MRI	18.2	ALL:80.6 Lung :69.4 Bone:25.8 Lymph node :8.1 Omentum:4.8 Adrenal:3.2	Steroids alone:40.3 SR: 9.7 WBRT alone :25.8 GKS:16.1 SR+WBRT:8.1	ALL:1.7 SC:0.5 Resection or WBRT or GKS:2.5 SR+WBRT:8.4	Single lesion, Child-Pugh's classification A Any treatment modalities for BM
Han et al.21 (2010)	Korea	1991-2007	NA	20	0.05	85	55	50	91	CT or/and MRI	18.5	85	GKS/WBRT/SRT:90 SC:10	ALL:2 WBRT and/or GKS:4 SR+adjuvant therapy:2	No ECMs, age < 60 years, no recurrent ICH
Uchino et al. 51 (2011)	Japan	1990-2006	2386	4	0.17	NA	NA	NA	NA	NA	NA	NA	Radiation:50; No Treatment:50	NA	NA
Hsiao et al.17 (2011)	Taiwan	1993-2006	NA	36	NA	80.5	56	NA	NA	CT or/and MRI	11.5	Lung:33.3 Bone:25	NA	1	NA
Jiang et al.3 (2012)	China	1994-2009	8676	41	0.47	80.5	48.5	58.5	46.3	CT/ MRI/PET-CT	15	80.5 Lung: 75.6 Bone: 22 Adrenal :9.8 other sites:7.3	SR or WBRT or and/or SRS:42.5 Steroid only:57.5	ALL:3 SR/WBRT/GKS:6.8 SC:2.7	no ECMs, low RPA class, any treatment modality for BM
Han et al. 86 (2013)	Korea	1998-2011	NA	32	NA	87.5	54	40.6	76.3	CT or/and MRI	26	96.9 Lung:87.5 Lymph node:31.3	SRS	2.8	Volume of BM,AFP
Han et al.2 (2013)	Korea	2001-2012	5015	33	0.65	90.9	62	52	52	CT or/and MRI	18.3	94 Lung :73 Bone:18 Lymph node:24 Adrenal:6 Skin:3	SR :12 SR + WBRT:18 GKS: 33 GKS + WBRT:6 WBRT: 12 Palliative :18	2.6 SR/SR+WBRT:6.3; GKS/WBRT/GKS+WBRT:2.6 steroid:0.25	SR, no ICH, Child-Pugh's classification A
Park et al.34 (2013)	Korea	2004-2012	NA	59	NA	83	52.2	NA	33.9	CT and MRI	NA	93.2	SC:28.8 GKS:33.9 SR:23.7 WBRT:13.6	ALL:1.1 SC:0.5 GKS:1.3 SR:3.7 WBRT:1.1	Active intervention for BM, RPA class, Child-Pugh's classification
Park et al.36 (2014)	Korea	1993-2012	NA	73	NA	87.7	52.5	56.2	47.9	MRI and/or CT	NA	ALL:93.2 lung :49.3 Bone:6.8 Lymph node :2.8 Multiple:34.3	GKS	4	age of ≤65 years, Child-Pugh Class A, KPS≥ 70, and low RPA class (I or II)
Lim et al. 12 (2014)	Korea	1995-2011	10,615	118	1.1	81.4	54	54.2	55.1	CT or/and MRI	NA	ALL:95 Lung:72.9 Bone :23.7 Lymph node:11.9 Omentum:3.4 Adrenal gland :5.9	Active treatment: 77 SC:41	ALL:1.5 Active treatment:2.6 SC:0.5	Number of BM, Child-Pugh-Class score, AFP level
Kim et al.35 (2014)	Korea	2000-2011	NA	95	NA	86.4	56.1	44.2	74.7	CT or/and MRI	29.5	92.6 Lung:88.4 Lymph node :33.7 Bone:14.7 Adrenal: 9.5 Others:6.3	Observation: 6.3% WBRT only: 60 RSy: 18.9 SR:3.15 RS +WBRT :2.1 SR+ WBRT: 6.3 SR+RS:3.2	ALL:3 Single or none: 2.61 Multimodality:10.56	Age, ECOG PS, Child-Pugh class, AFP level, controlled primary tumor status, number of BM
Xu et al.4 (2014)	China	2011-2013	NA	14	NA	85.7	53	57.1	59.1	NA	26	Lung:57.1 Bone:35.7 Both:21.4	GKS	5	Total volume of BM, RPA class and AFP level
Kim et al.50 (2015)	Korea	2000-2013	NA	105	NA	84	56.3	45.7	79	CT or/and MRI	29.5	93.8 Lung:91.4 Lymph node :28.4 Bone:11.1 Adrenal: 8.6 Others:6.2	WBRT only: 58.0 SR+ WBRT: 8.6 RS +WBRT :3.7 SR:3.7 RS:22.2 SR+RS:3.7	ALL:3 Single or none: 2.61 Multimodality:10.56	Age, ECOG PS, Child-Pugh class, AFP level, controlled primary tumor status, number of BM
Park et al.53(2015)	Korea	2000-2013		97	NA	85.57	56.6	37.1	69.1	CT or/and MRI	NA	95.9 Lung:94.8 Lymph node :27.8 Bone:17.5 Adrenal: 9.3 Others:14.4	WBRT: 73.2 surgery/radiosurgery + WBRT: 18.6 WBRT as salvage :8.2	ALL:3.5 WBRT:1.1 GKS:1.3 SR:3.7 SC:0.5	ECOG PS, Child-Pugh classification, AFP level, treatment aim
Yamakawa et al.31 (2015)	Japan	2004-2012	1702	15	0.9	66.67	64	NA	66.7	CT or/and MRI	17.8	Lung:73.3	WBRT:66.7 GKS:33.3 SRT:26.7	RT:5 SC: 0.6	ICH,RT
Kato et al.105 (2015)	Japan	2011-2015	NA	7	10.8	NA	NA	NA	NA	NA	NA	NA	Cyber-knife	NA	NA

Abbreviations: BMHCC: Brain metastases from hepatocellular carcinoma, Inc: Incidence of brain metastases, ECM: Percentage of patients diagnosed with extra-cranial metastases, OS: Overall survival, ECOG: Eastern Cooperative Oncology Group, PS: Performance status, KPS: Karnofsky performance status, ICH: Incidence of intracranial hemorrhage, SR: Surgical resection, SC: Supportive care; RT: Radiotherapy, SRT: Stereotactic radiotherapy, SRS: Stereotactic radiosurgery, WBRT: whole brain radiotherapy, GKS: Gamma knife surgery, RPA: Recursive Partitioning Analysis, NA: Not available

## CHARACTERISTICS OF BMHCC PATIENTS

### Formation and timing of brain metastases

**Figure 1:**
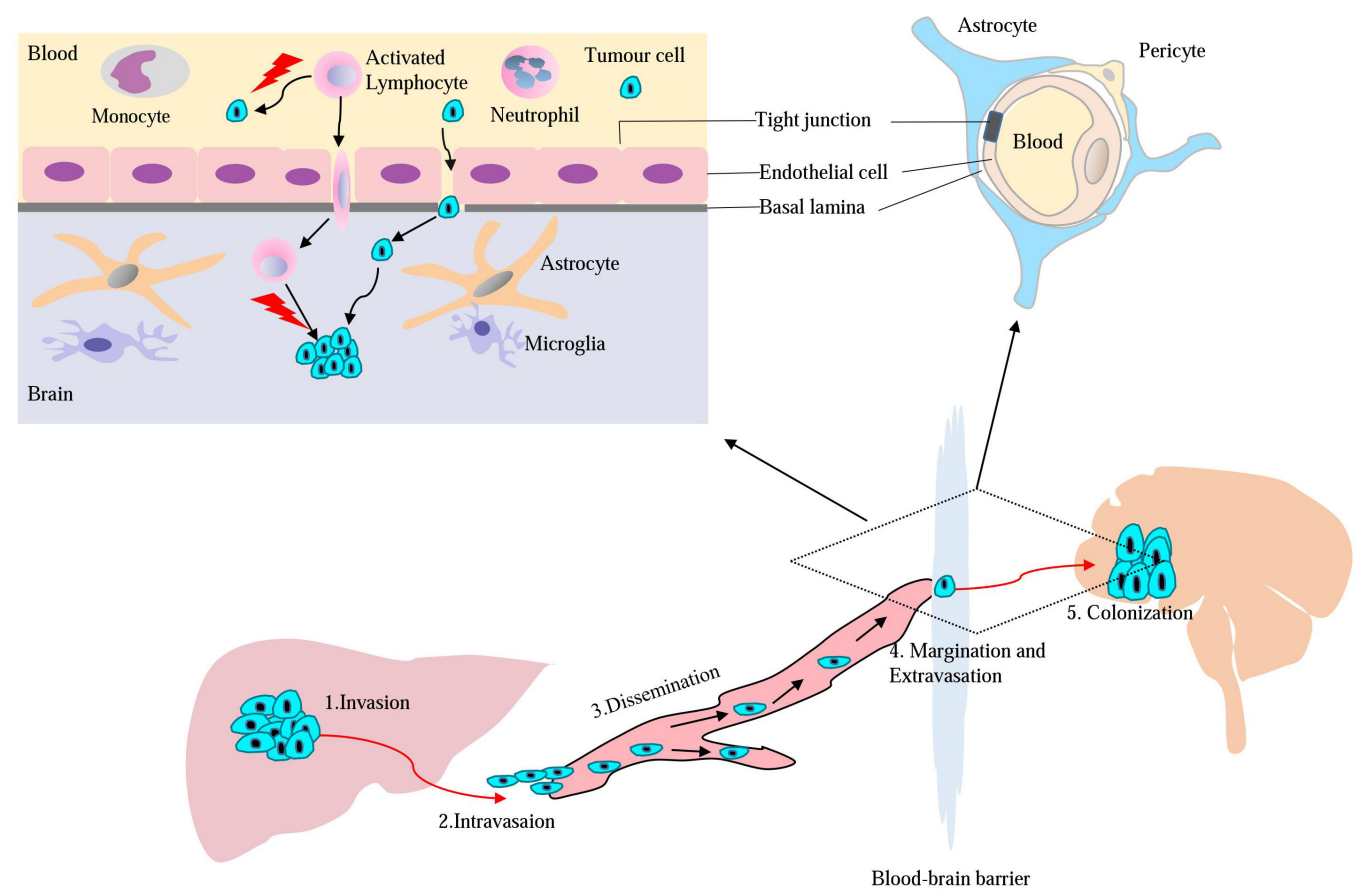
Model for major mechanisms of HCC metastasis to the brain and T cell pass through blood-brain barrier (BBB)

Brain metastases tend to occur in patients with advanced HCC and distant metastasis is a multistep process, often referred to as metastatic cascade (Figure 1) [21, 22]. As shown in Table 1, the interval from initial HCC diagnosis to discovery of brain metastasis was between10.5 and 29.5 months. Importantly, most of the published series reported an interval <20 months, which is shorter than that patients with brain metastases from lung cancer, breast cancer and melanoma (Table 2) [23–29].

**Table 2:** Comparison of the clinical characteristics of brain metastases from different tumors

Primary tumor	Pro (%)	Inc (%)	Single BM (%)	ECM (%)	Time since primary cancer diagnosis (months)	OS (months)
Lung cancer	40-50^8,9^	30-50^23,39,57,60^	42-45^37,40,45^	15-69^23,38-40^	24-33^23,24^	3-7^57,58,60^
Breast cancer	15-25^8,9^	10-30^26,46^	20-43^41,42,46^	14-83^27,41,42^	32-39^25-27^	2-16^26,42,55^
Melanoma	5-20^8,9^	17-45 ^43,60^	13-29 ^28,43,47,48^	45-66^37,43^	24-48^28,29^	3-6^59-61^
HCC	NA	0.2-2.2^10-15^	37.1-90.0^16,53^	80.5-96.9^3,86^	10.5-29.5^35,49^	1-3^17,50,86^

Abbreviations: HCC: Hepatocellular carcinoma, Pro: Proportion of brain metastases from a specific tumor, Inc: Number of patients of brain metastases from a specific tumor/ Number of the specific tumor patients, ECM: Percentage of patients diagnosed with extra-cranial metastases, OS: Overall survival, NA: Not available

### Metastatic disease

BMHCC patients generally have advanced liver disease and a high percentage of synchronous extra-cerebral metastases (ranging from 80.5% to 93.2%), including lung (69.4-75.6%), followed by bone (18-25.8%), lymph nodes, and adrenal glands [2, 3, 12, 30–36]. On the other hand, BMs from early stage HCC or without other extrahepatic metastases are occasionally reported [30]. Moreover, the majority of the available studies reporting that 70 % or more patients had lung metastases. In line with other reports, Seinfeld et al. noted that BM from HCC could be secondary to lung deposits [14, 31, 33]. Therefore, BM should be suspected for patients with extracranial metastasis, especially for metastasis to the lung. Typically, there is a considerable systemic tumor burden in BMHCC patients. Furthermore, the prevalence of extracranial metastases in BMHCC is higher than the brain metastases from lung cancer, breast cancer and melanoma (Table 2), possibly reflecting a heavier tumor burden among BMHCC patients [27, 37–43].

### Number of brain metastases

Compared with BMs from other types of cancer, most patients with BMHCC have solitary intracranial metastasis (Table 1). The parietal lobe is the most common brain metastasis site, followed by the frontal lobe [3, 17]. Several studies reported a prevalence of single BM from various BMHCC populations, ranging from 37.1 % to 90.0 %, and most of the studies reported an incidence of more than 50%. By contrast, BMs from lung cancer, breast cancer and melanoma generally had multiple brain lesions (Table 2) [37, 40–42, 44–48].

### Clinical presentation

The neurologic manifestations of HCC include hepatic encephalopathy, paraneoplastic syndromes, and metabolic encephalopathy [17]. The most frequent symptom is hepatic encephalopathy caused by elevated serum ammonia level [49]. In contrast, paraneoplastic syndrome is not common in patients with BMHCC. A specific neurologic paraneoplastic syndrome from HCC is cerebellar atrophy [49]. Similar to BM from other cancers, there are also some nonspecific symptoms resulting from BM, including intracranial hypertension (space-occupying mass), headache, focal neurological symptoms, and altered consciousness.

By contrast, a remarkable and interesting finding is that patients with BMHCC commonly experience intracerebral hemorrhage (ICH), with the incidence ranging from 33.9% to 70% [3, 21, 31, 33, 34, 50]. Given this, sudden onset of symptoms often heralds life threatening ICH. Some experts hold that the hypervascularity of HCC and underlying coagulopathy may explain this observation [33, 34, 51]. ICH usually causes sudden neurologic deterioration and leads to severe neurologic deficits, thus resulting in poor prognosis [31]. However, there is controversy over the effect of ICH on survival among BMHCC patients. Han et al. reported that patients without ICH had longer survival than patients with ICH (13.7 weeks vs. 8.1 weeks) [2]. On the contrary, Hsieh et al. [52] and Jiang et al. [3] reported that the presence of ICH did not influence the overall survival of patients with BMHCC. More researches are needed to resolve this controversy and explore the underlying mechanisms.

## NATURAL COURSE

Brain metastasis in HCC patients represent a catastrophic event that portends a uniformly poor prognosis with studies reporting 1- and 5-year survival rates of 2.7% and <5%, respectively [18, 21, 33]. With best supportive care only, median survival is just around two weeks [12, 20, 31, 33, 53, 54]. Even with treatment, the prognosis for these patients remains poor with a median survival time that ranges from 4 to 12 weeks (Table 1), with mortality due largely to systemic disease progression or metastatic brain disease [17, 33, 35, 52]. This survival seems more dismal than BM from lung cancer, breast cancer and melanoma [55–61]. Encouragingly, in some circumstances, survival could be extended to 12 months after comprehensive treatment [30]. Therefore, a relatively better prognosis for patients with BMHCC may be achieved after comprehensive therapy [21].

## PROGNOSTIC FACTORS

Patients with BMHCC usually have poor prognosis and low quality of life (QOL). Many factors, including primary HCC characteristics and clinical variables, could help predict prognosis [3, 12, 33, 35, 53]. Such variables include performance status (PS), systemic disease status, alpha-fetoprotein (AFP) level, Child-Pugh classification, number of BMs, and treatment modality. AFP reflects the tumor burden and the Child-Pugh classification reflects liver function: these two factors are HCC-specific and their prognostic values have been confirmed in several previous studies [2, 12, 33, 35, 53]. The number of brain lesions is also an important prognostic factor. Some investigators reported that the median survival of patients with single brain lesions appears to be longer than that of patients with multiple lesions [2, 12, 33, 35, 53]. Additionally, the impact of the presence of extra-cranial metastases on survival remains unclear [3, 21, 33] and further studies are needed to elucidate it.

To predict the prognosis of patients with BMHCC, several prognostic scores including recursive partitioning analysis (RPA), Graded Prognostic Assessment (GPA), Basic Score for Brain Metastasis (BSBM), and Score Index for Radiosurgery (SIR), have been devised [62, 63]. The most widely used indices over the last decade were RPA classes and GPA. In the late 1990s, the Radiotherapy Oncology Group (RTOG) first performed a recursive partitioning analysis (RPA) classification including age, Karnofsky Performance Score (KPS), status of primary tumor, and extra-cranial disease. This analysis separated patients into three different classes (RPA classes I, II, and III) (Table 3) [64]. Patients in class I or II showed a longer survival than those in RPA class III (6 months vs. 1 month, P < 0.0001). As RPA does not incorporate the number of brain metastases and some subjective components are difficult to quantify, RTOG subsequently developed a more objective prognostic index called the graded prognostic assessment (GPA) scoring system [53, 64, 65]. Considering that the prognostic factors for BM vary according to primary diagnosis, the original GPA was subsequently modified to diagnosis-specific Graded Prognostic Assessment (DS-GPA) [65]. However, to date only three DS-GPA indices have been developed for BMHCC [12, 35, 53]. In 2014, two studies conducted in Korea focused on BMHCC (summarized in Table 4). Kim et al. [35] assigned patients into three groups according to Eastern Cooperative Oncology Group performance status (ECOG PS), Child-Pugh class, AFP level, and number of brain lesions. Another study conducted by Lim et al. [12] assigned a score from 0 to 4 based on number of brain metastases, AFP, and Child-Pugh grade. This score system was independently validated in a cohort of 25 patients. One year later, a nomogram taking into account four variables (ECOG PS, Child-Pugh classification, AFP, and treatment aim) was developed [53]. Compared with other prognostic indices, the nomogram provides an individualized estimate of survival with a high concordance probability and may help more precise and individualized management of patients [66].

**Table 3:** Prognostic scores used for patients with brain metastases

Score	Age (years)	Performance status	Number of brain metastases	Extra-cranial metastases	Controlled Primary	Class I	Class II	Class III	Class IV
RPA Derived from 3 prospective RTOG studies, n = 1,200	age <65	KPS≥70vs <70	NA	Present or Absent	Yes or No	All 4 factors favorable (7.1 mos)	other patients	KPS <70 (2.3 mos)	NA
GPA Derived from 5 prospective RTOG studies, n = 1,960	<50:1 Point 50-60: 0.5 points >60: 0 points	KPS 90-100: 1 point KPS 70-80: 0.5 points KPS <70: 0 points	1: 1 point 2-3: 0.5 points >3: 0 points	Present: 0 points Absent: 1 point	NA	3.5-4 points (11 mos)	3 points (8.9 mos)	1.5-2.5 points (3.8 mos)	0-1 Points (2.6 mos)

Abbreviations: BM: Brain metastases, GPA: Graded prognostic assessment, KPS: Karnofsky performance status, RPA: Recursive partitioning analysis, NA: Not applicable

**Table 4:** Two HCC-specific graded prognostic assessment (HCC-GPA) indexes for BMHCC patients

Study	Performance status	Child-Pugh Class	Number of brain metastases	AFP(ng/ml)	Class I	Class II	Class III	Class IV
Kim et al.35	ECOG >2 vs≤2	A vs B/C	Single vs Multiple	<1,400 vs >1,400	0-1 risk factor (5.8 months)	2 risk factors (2.5 months)	3-4 risk factors (0.6 months)	NA
Lim et al.12	NA	A: 3 points B: 2 points C: 0 points	Single:0.5 points Multiple:0 point	<400:0.5 points >400:0 points	4.0 points(27weeks)	3.0-3.5 points(7.9weeks)	1.5-2.5 points(3.2weeks)	0-1 points(1.7weeks)

Abbreviations: BMHCC: Brain metastases from hepatocellular carcinoma, GPA: Graded prognostic assessment, ECOG: Eastern Cooperative Oncology Group, NA: Not applicable

It should be noted that RPA and GPA are not diagnosis specific and were not validated in BMHCC patients. Furthermore, HCC is a very heterogeneous tumor, therefore further studies are warranted to verify their clinical application in BMHCC patients. The three HCC-GPA models were all developed from small-scale retrospective studies rather than a prospective randomized trial. Because of the weak statistical power, further studies to verify and develop new models are needed. Nonetheless, these tools may still help clinicians stratify patients and select appropriate therapy for BMHCC patients.

## DIAGNOSIS OF BMHCC

Because BMHCC usually carry a substantial morbidity and mortality rate, therefore early diagnosis is very important for improving the prognosis. Computed tomography (CT) and magnetic resonance imaging (MRI) are the two most commonly used imaging modalities for detecting brain lesions [67, 68]. The use of CT as a central nervous system (CNS) imaging modality has gradually declined over the last 30 years and has been largely replaced by MRI [68]. MRI can detect most of BMs and its sensitivity and specificity is markedly greater than that of CT, particularly when the lesions are very small or located in the posterior fossa [46, 69, 70]. MRI may be useful for differential diagnosis between primary brain tumors and BM, and careful staging can often detect a peripheral source of BM so brain biopsy is usually unnecessary [69]. Generally, MRI is recommended as the first-line imaging modality for potential BM [71, 72]. However, although MR imaging is the preferred imaging modality, CT remains a vital tool for initial work-up and perioperative management [73]. It is extremely useful for patients who present with new focal deficits because it is easily performed, well tolerated, and can rapidly rule out life-threatening emergencies such as hemorrhage, hydrocephalus, and significant mass effect [73]. Given the propensity of this vascular tumor to hemorrhage, CT may play a more important role in BMHCC patients than in BMs from other cancers. The role of routine screening of asymptomatic HCC patients for the development of BM by imaging remains controversial since no study has investigated whether effective screening can prolong overall survival (OS) and prevent serious symptoms [70]. Studies focusing on this issue are urgently needed.

## MANAGEMENT OF BMHCC

### Principles and goals of treatment

Because of the rarity and poor prognosis of BMHCC, guidelines on diagnosis and therapeutic strategy have not been established. The treatment approach for BMHCC may be similar to general guidelines for metastatic brain tumors [2]. Optimal treatment requires an individualized process for each patient, depending on the patient's clinical status (e.g., neurologic deficit, Child-Pugh class, life expectancy), disease burden (AFP level, extracranial disease control, especially the number of BMs), and characteristics of the primary HCC (radiologic aspect, size, location) [12, 53, 74]. Therefore, an integrative multidisciplinary approach for each case from diagnosis to treatment is always recommended [62, 74]. Current options include surgery, whole-brain radiation therapy (WBRT), stereotactic radiosurgery (SRS), chemotherapy, targeted agents, immunotherapy, and supportive measures. With respect to treatment goals, for patients with favorable prognostic factors who might benefit from an aggressive treatment, the goal of treatment is to prolong survival and improve QOL, whereas for those with short survival expectancy, stabilizing BMs and palliating symptoms is warranted [74].

### Local treatment of BMHCC

WBRT, SRS, and surgical resection are the current options for treatment of BM. Unfortunately, BMHCC usually occurs in cases of advanced HCC and the patients tend to show a considerable deterioration in PS. Consequently, locoregional treatment may not lead to a clinical benefit and/or gain in survival.

### Surgical resection

National guidelines recommend that surgery should be considered for the management of BM patients with single or few (≤3) lesions, particularly when the systemic disease is well controlled and the BMs are symptomatic [72, 75]. Surgical resection not only can lead to an immediate elimination of life-threatening status and symptom-generating mass effect, but also a reduction of focal neurologic deficits and a rapid steroid taper [5, 74]. Compared with best supportive care, surgical metastectomy has been shown to provide a significantly prolonged survival benefit (>3 months vs. <2 weeks) in BMHCC patients in multiple retrospective series [2, 33, 34]. Surgery is recommended in cases with a limited number of lesions (≤3), younger patients, and those with good KPS [36, 72, 75]. However, surgery is also recommended to palliate symptoms in cases with a single large metastasis (>3 cm) and a significant mass effect (1-cm midline shift). It is generally thought to improve QOL despite the lack of a survival benefit [26, 36]. Although more than half of patients presented with single intracranial metastasis [10, 17, 21, 49], only a small proportion (<15%) underwent surgical resection because of considerable liver function deterioration and other medical problems [2, 33, 35]. Nevertheless, surgical resection remains an appropriate therapeutic option for eligible patients with BMHCC.

## RADIOTHERAPY

### WBRT

WBRT has been used as the principal treatment for multiple (≥4) brain metastases or as an adjuvant treatment [20, 76]. Considering the acute adverse effects (fatigue, alopecia, Eustachian tube dysfunction) and late neurotoxicity associated with WBRT, this approach is usually not recommended unless necessary [77]. Historically, WBRT alone is used to provide relief of symptoms in patients for whom surgery and SRS are contraindicated [26], although it may not alter the survival of these patients [62, 78]. Recently a phase III randomized trial suggested that WBRT provides little clinical benefit compared to supportive care for patients with non-small cell lung cancer (NSCLC) and BM who are aged >60 years and have KPS <70 [79], therefore WBRT should be used with great caution.

### Adjuvant WBRT

There is still controversy over whether adjuvant WBRT results in a clinical advantage for patients, especially those with poor prognosis [63, 72]. Most studies (reviewed in [26, 62, 72, 78, 80, 81]) have clearly demonstrated that the addition of WBRT to surgery or SRS was associated with improved local control rates and decreased neurologic deaths but did not improve outcome in terms of survival (class I evidence). With respect to BMHCC, a retrospective study by Choi et al. [33] concluded that resection in conjunction with WBRT showed considerably prolonged survival (33.6 weeks). Despite the lack of a survival benefit, it is now generally accepted that adjuvant WBRT can be considered in the case of absent/controlled systemic disease and good PS (KPS >70) [72]. Considering the established potential adverse effects of WBRT, omission of WBRT appears to be attractive in patients with a limited number of BM [80]. Moreover, it is noteworthy that a retrospective study from Japan showed that radiotherapy could prevent intracranial hemorrhage and improve survival [31]. Recently, Kim et al. [50] also concluded a similar result through retrospective analysis though statistical significance was not reached. As mentioned above, BMHCC is frequently to be accompanied by ICH, whereas optimal management of this scenario has not been established. Given the peculiarity of BMHCC, WBRT appears to be an important strategy to improve survival through preventing ICH. However, more studies are needed to offer definitive conclusions.

### Stereotactic radiosurgery

SRS permits the delivery of high doses of radiation to a small target (<3.5 cm) using gamma-knife (GK) or a linear accelerator (Linac) [72, 77]. SRS has become an increasingly popular treatment option for patients with 1-4 metastases [81], and can be considered as an alternative for surgery or a salvage treatment in patients who are not candidates for surgery [74, 82–84]. Typically, when the metastases are larger than 3-4 cm or immediate mass relief is required, surgery is preferred [63, 74]. Recently, postoperative SRS to the surgical cavity is becoming popular since it could maximize local control. In contrast to WBRT, SRS might avoid late neurocognitive effects [77]. However, only a limited number of retrospective studies have specifically assessed radiotherapy of BMHCC (summarized in Table 5). Of note, due to the risk of CNS radiation toxicity in accordance with the RTOG 90-05 dosing guideline, most reports suggest a prescribed mean dose of <20 Gy, whereas the estimated biologically effective dose for HCC tumor is approximately 20 Gy or more (Table 5). A few studies have demonstrated that the marginal dose more than 20 Gy appears to be sufficient to control BMHCC and preserve neurologic function [4, 85, 86]; however, the evidence is not solid and the optimal dose is not yet clearly defined.

**Table 5:** Summary of published studies of radiosurgery for the treatment of BMHCC

Study (year)	Study design	Cases (n/a)	Mets (n)	Mean Tumor Volume cm3 (range)	Margin Dose in Gy (range)	Radiosurgery regimen(s)	Local control (%)	Median OS(weeks)
Chang et al.[49](2004)	Retrospective	1/45	NA	NA	NA	NA	NA	>16
Hiraoka et al.[85] (2008)	Retrospective	1	1	NA	35*	Cyber-Knife	100	NA
Choi et al.[33] (2009)	Retrospective	10/62	NA	NA	13.5 (10-15)	GKS	NA	10
Han et al.[21] (2010)	Retrospective	12/20	34	NA	NA	GKS	NA	16
Jiang et al.[3] (2012)	Retrospective	9/41	NA	NA	16 (14-20)	NA	NA	13.5
Han et al.[2] (2013)	Retrospective	13/33	NA	NA	18 (14-25)	GKS	NA	10.4
Han et al.[86] (2013)	Retrospective	32	80	6.1 (0.01-67.3)	20.1 (10-25)	GKS	51.3	11.3
Xu et al.[4] (2014)	Retrospective	14	22	8.2 (0.59-27)	18.7 (10-22)	GKS	NA	20
Park et al.[36] (2014)	Retrospective	73	141	7.3 (0.19-33.7)	23 (15-32)	GKS	79.6	16
Kato et al.[105] (2015)	Retrospective	NA	7	23.5 (12-38)†	22 (14-30)	GKS	28.6**	NA
Yamakawa et al.31 (2015)	Retrospective	7/15	NA	30 (5-40)†	NA	GKS,SRT	NA	22.4

Abbreviations: BMHCC: Brain metastases from hepatocellular carcinoma, n/a: Number of patients treated with radiosurgery/ Total number of BMHCC patients, Mets: metastases, OS: Overall survival, RS: Radiosurgery, SRT: Stereotactic radiotherapy, GKS: Gamma knife surgery, NA: Not available, *A margin dose of 35 Gy in 5 fractions was used, †Median size(mm), * *Complete response rate

### Cyber-knife (CK)

Cyber-knife (CK), a robotic image-guided system, has been successfully used in many types of tumor including brain cancer, liver cancer, and other types of cancer[87–90]. In 2008, Hiraoka et al. [85] first reported the use of cyber-knife for BMHCC, and showed that it provided excellent local control with acceptable toxicity and could also prevent re-hemorrhage. Compared with GK, CK is a relatively non-invasive treatment modality. It could provide more accurate target localization, higher tumor control, and lower toxicity with repeated treatments for recurrent metastases [88, 91]. To date, there is robust evidence supporting the efficacy of CK in treating BM from different primary cancers. Thus, CK appears as a promising strategy for treating BMHCC patients, not yet sufficiently supported by convincing clinical data in this population.

### Cytotoxic chemotherapy

HCC is known to be a relatively chemotherapy-resistant tumor. Conventional cytotoxic chemotherapy has not been proved to prolong the overall survival for any subset of HCC [92, 93]. The choice and efficacy of chemotherapy depend on chemosensitivity of the primary tumor. In addition, the brain is difficult to access for chemotherapeutic drugs due to limited penetration through the BBB [20, 94]. Therefore, chemotherapy may be not suitable for treatment of BMHCC.

### Supportive care

The survival of BMHCC patients is poor, with a mean survival period of 3 months. Patients with poor prognosis should be managed supportively [79, 95, 96]. Although therapies and technologies have improved a lot, in past studies at least 18-40% of BMHCC patients received conservative care [2, 12, 33, 34]. Thus, effective supportive treatment, including glucocorticoids, antiepileptic drugs (AEDs), and anticoagulant medications, is critical for BMHCC patients and could improve QOL [77, 97].

As reported in available literatures, the majority of cases of BM with cerebral edema have been managed with glucocorticoids, which could improve related symptoms[72, 76]. Dexamethasone is generally considered the steroid of choice, mainly because of its relatively low mineralocorticoid activity, long half-life, and decreased risk of cognitive impairment [76, 77]. A marked neurologic improvement is expected within 24-72h after dexamethasone use in up to 75% of patients with brain metastases [98]. The recommended starting dose depends on the severity of symptoms. Guidelines support an initial dexamethasone dose of 4-8 mg per day in two divided doses; however, these recommendations are based on level 2-3 evidence [72, 98]. For severe symptoms related to increased intracranial pressure (ICP), a starting dose of 16 mg/d or more should be considered [98]. As dexamethasone is effectively eliminated by hepatic mechanisms and up to 40% of BMHCC patients have poor liver function (Child-Pugh classification B/C) [3, 33, 35], so dosage adjustment may be required in this group of patients.

Patients with seizures should be treated with standard AEDs. There is no evidence that prophylactic use of AEDs can prevent future seizures, therefore it is not recommended in asymptomatic patients [72, 74, 76, 77]. As short-term prophylactic AED use with rapid tapering off reduces the risk of seizures by 40-50% within the first postoperative week, the American Association of Neurology recommends tapering of prophylactic AEDs after the first postoperative week in the perioperative setting [99].

Venous thromboembolism (VTE) is common in cancer patients, especially in patients with brain tumors. It is considered as the second leading cause of death among this population [100, 101]. The management of BMHCC patients is particularly challenging because of the high risk of ICH and poor liver function, and no data are available on the clinical scenario. A single-center retrospective study demonstrated that anticoagulation did not significantly increase the risk of intracranial hemorrhage for melanoma patients with hemorrhagic BM and guidelines recommend anticoagulant treatment for patients with brain metastases [72]. However, there is no evidence regarding the safety of anticoagulation therapy in BMHCC patients and use of anticoagulants in BMHCC merits careful consideration.

### Novel treatments

Although many patients may not be suitable for resection or radiotherapy, some novel treatments show great promise for the management of BMHCC.

### Targeted therapy

Targeted therapy has recently emerged as an important modality for the treatment of BM from different types of tumors. Several prospective trials have shown unequivocal clinical activity in melanoma and NSCLC (reviewed in [77]). Many trials are focusing on molecular targeted agents in HCC, but most have ended in failure except sorafenib [102, 103] and even this agent shows only a limited increase in survival of approximately 3 months for advanced HCC [104]. Disappointingly, however, BM is a contraindication for sorafenib [105]. In addition, because of the diversity of the carcinogenesis mechanisms of HCC, most molecular targeted agents provide only partial inhibition of signaling pathways [106–109] and combinatorial approaches with other therapies may be more appropriate.

### Immunotherapy

Immunotherapy is emerging as a promising treatment for intractable cancers such as hematologic malignancies, melanoma, and other solid cancers [82, 110, 111]. Among immunotherapies, adoptive cell therapy (ACT) and immune checkpoint blockade are hot research areas.

### Adoptive cell therapy (ACT)

ACT is a highly personalized form of passive immunization in which tumor cells are destroyed following the infusion of autologous or redirected tumor-specific T cells [112]. ACT has shown encouraging therapeutic efficacy in the treatment of a variety types of cancers including brain metastases [112–114]. Moreover, many clinical trials also have demonstrated that ACT showed promising antitumor effects against HCC. Recently, a review conducted by Liu et al. [115] concluded that about half of immunotherapy clinical trials in HCC involve ACT and its use can significantly improve the recurrence and survival of HCC. Preliminary results of a recently published phase I trial showed that ACT was safe and most of the patients had no disease progression at a median follow-up of 14 months [116]. Although there is no research available concerning the effect of ACT in BMHCC, ACT has been proved to eradicate metastatic tumor cells [111]. Recent advances demonstrated that ACT was capable of abrogating brain metastases from melanoma with complete and durable responses [114, 117]. Because several studies have demonstrated that activated T cells could pass through the BBB and remain active (Figure 1) [118, 119], based on the satisfactory effects in BMs and HCC we can hypothesize that ACT may be effective in BMHCC. Ongoing and future research studies are expected to provide more data on this topic.

### Immune checkpoint blockade

**Figure 2:**
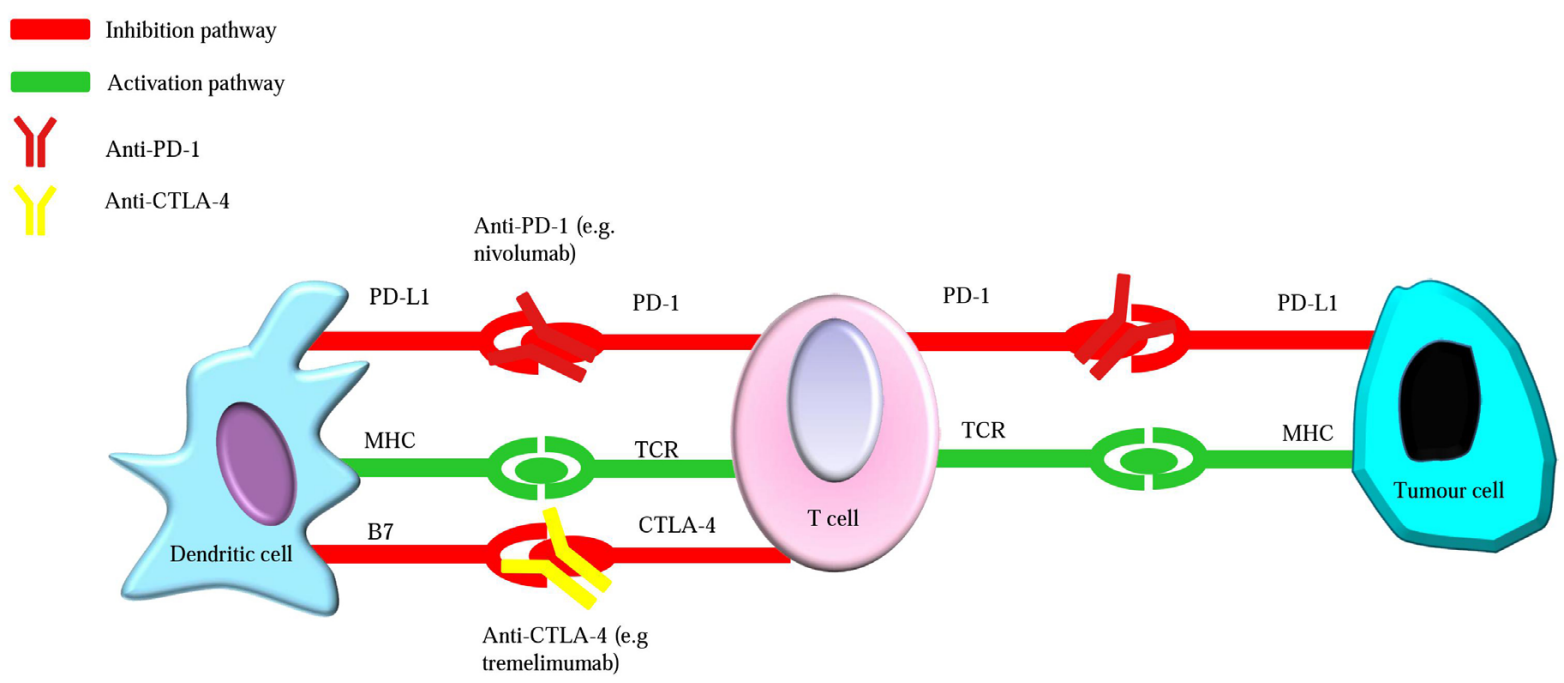
Simplified mechanism of CTLA-4 and PD-1 inhibitors in tumor immunotherapy T cell activation leads to the up-regulation of immune checkpoint molecules such as CTLA-4 and PD-1 which act to abrogate T cell responses. Anti-CTLA-4 and anti-PD-1 antibodies reverse the immunosuppressive effect when they bind CTLA-4 receptors and PD-1 on T cells, respectively. CTLA-4, T-lymphocyte antigen-4; PD-1, programmed death 1; PD-L1, programed death-ligand-1; MHC, major histocompatibility complex; TCR, T cell receptor. Notably, the scheme is highly simplified: in reality CTLA-4 and PD-1 act through multiple mechanisms.

The balance between co-stimulatory and co-inhibitory signals, defined as “immune checkpoints”, determines cytotoxic T-cell activation and the intensity of the immune response [120]. T-lymphocyte antigen-4 (CTLA-4) and programmed death 1 (PD-1) are two of most prevalent immune checkpoint proteins. The mechanisms of CTLA-4 and PD-1 and their possible roles in treating HCC were recently reviewed (Figure 2) [121]. The CTLA-4 inhibitor tremelimumab showed a favorable safety profile and significant antitumor effects against advanced HCC, with a 76% disease control rate in a phase I clinical trial [122]. A phase I/II clinical trial is currently ongoing to test the safety and antitumor activity of an anti-PD-1 antibody (nivolumab) for advanced HCC (NCT01658878). The interim findings were presented at the 2016 ASCO Annual Meeting [123]. Investigators demonstrated that the treatment was well tolerated with a manageable safety profile and the estimated survival rate in evaluable patients (n=48) was 59% at 12 months. Another phase III study to determine whether nivolumab or sorafenib is more effective in the treatment of advanced HCC was initiated in 2015 and is currently ongoing (NCT02576509). The initial findings were announced at the 2016 International Liver Cancer Association Annual Conference, showing an encouraging 9-month overall survival rate of 71% (n=214) with a favorable safety profile [124]. In addition, immune checkpoint inhibitors have shown impressive clinical efficacy in brain metastases from melanoma and NSCLC in early clinical trials [125–131]. Based on these results, we have great confidence for future use of checkpoint blockers in the treatment of BMHCC. Furthermore, accumulating preclinical and observational data suggest that the integration of immunotherapy and radiation therapy has notable clinical efficacy in brain metastases [128, 132]. Further clinical results will help elucidate the role of this novel combination.

## CONCLUSIONS & FUTURE PERSPECTIVES

Brain metastases represent a critical stage of HCC and the frequency is expected to increase. Therefore, it is increasingly important for physicians to understand its characteristics, implications, and potential treatments. Comparing with BM from other types of cancer, BMHCC tend to confer a worse prognosis due to heavier systemic tumor burden, severe underlying liver dysfunction and much higher incidence of ICH. Consequently, a considerable proportion of BMHCC patients are not eligible for any treatment but supportive care. Given the high prevalence of ICH, the using of anticoagulants in BMHCC patients merits careful consideration and there is an urgent need for effective therapies to prevent ICH. Based on current evidence, in cases with a limited number of lesions (≤3), good prognostic factors, and controlled systemic disease, surgery is recommended. WBRT remains a standard option for patients with limited performance status and/or multiple metastases, may reduce the incidence of ICH and warrants further clinical investigation. The development of CK has also contribute to local control of this disease. Chemotherapy and targeted therapy have limited efficacy on BMHCC. Notably, immunotherapy has the potential to achieve complete, long-lasting remissions and cancer cures, representing the most promising cancer treatment approach. Currently, combinatorial therapy is considered as a promising strategy for cancer treatment. Nonetheless, the optimal sequence and/or combination of the available treatment modalities requires further exploration.
